# Long-Term Hypercaloric Diet Consumption Exacerbates Age-Induced Dysmetabolism and Carotid Body Dysfunction: Beneficial Effects of CSN Denervation

**DOI:** 10.3389/fphys.2022.889660

**Published:** 2022-05-04

**Authors:** Bernardete F. Melo, Joana F. Sacramento, Adriana M. Capucho, Dinis Sampaio-Pires, Cláudia S. Prego, Silvia V. Conde

**Affiliations:** CEDOC, CEDOC, NOVA Medical School, NMS, Faculdade de Ciências Médicas, Universidade NOVA de Lisboa, Lisboa, Portugal

**Keywords:** carotid body, metabolic disease, ageing, hypercaloric diets, carotid sinus nerve, insulin receptor

## Abstract

Carotid bodies (CBs) are metabolic sensors whose dysfunction is involved in the genesis of dysmetabolic states. Ageing induces significant alterations in CB function also prompting to metabolic deregulation. On the other hand, metabolic disease can accelerate ageing processes. Taking these into account, we evaluated the effect of long-term hypercaloric diet intake and CSN resection on age-induced dysmetabolism and CB function. Experiments were performed in male Wistar rats subjected to 14 or 44 weeks of high-fat high-sucrose (HFHSu) or normal chow (NC) diet and subjected to either carotid sinus nerve (CSN) resection or a sham procedure. After surgery, the animals were kept on a diet for more than 9 weeks. Metabolic parameters, basal ventilation, and hypoxic and hypercapnic ventilatory responses were evaluated. CB type I and type II cells, HIF-1α and insulin receptor (IR), and GLP-1 receptor (GLP1-R)-positive staining were analyzed by immunofluorescence. Ageing decreased by 61% insulin sensitivity in NC animals, without altering glucose tolerance. Short-term and long-term HFHSu intake decreased insulin sensitivity by 55 and 62% and glucose tolerance by 8 and 29%, respectively. CSN resection restored insulin sensitivity and glucose tolerance. Ageing decreased spontaneous ventilation, but short-term or long-term intake of HFHSu diet and CSN resection did not modify basal ventilatory parameters. HFHSu diet increased hypoxic ventilatory responses in young and adult animals, effects attenuated by CSN resection. Ageing, hypercaloric diet, and CSN resection did not change hypercapnic ventilatory responses. Adult animals showed decreased type I cells and IR and GLP-1R staining without altering the number of type II cells and HIF-1α. HFHSu diet increased the number of type I and II cells and IR in young animals without significantly changing these values in adult animals. CSN resection restored the number of type I cells in HFHSu animals and decreased IR-positive staining in all the groups of animals, without altering type II cells, HIF-1α, or GLP-1R staining. In conclusion, long-term hypercaloric diet consumption exacerbates age-induced dysmetabolism, and both short- and long-term hypercaloric diet intakes promote significant alterations in CB function. CSN resection ameliorates these effects. We suggest that modulation of CB activity is beneficial in exacerbated stages of dysmetabolism.

## Introduction

Ageing is a biological process characterized by a progressive deterioration in physiological functions and metabolic processes that drive morbidity and mortality (Barzilai et al., 2012; López-Otín et al., 2013). Metabolically, ageing is characterized by insulin resistance, changes in body composition, and decreased growth hormone and insulin-like growth factor-1 levels, among others, a process that is altered or accelerated when metabolic and cardiovascular diseases are present (Bonomini et al., 2015). In fact, it is recognized that diabetes and obesity cause the acceleration of cellular processes associated with ageing (López-Otín et al., 2013), such as deterioration of the structure and function of organs due to oxidative stress, increased inflammation, genetic instability, and disturbance of homeostatic pathways (Barzilai et al., 2012; Tzanetakou et al., 2012; Bonomini et al., 2015).

Carotid bodies (CBs) are chemoreceptor organs that sense alterations in arterial blood gasses such as hypoxia and hypercapnia and acidosis. These stimuli activate the CBs leading to the release of neurotransmitters that act on the carotid sinus nerve (CSN) to increase or inhibit its activity (Gonzalez et al., 1994). The action potentials generated postsynaptically are integrated in the brainstem to induce cardiorespiratory responses, normalize blood gases *via* hyperventilation (Gonzalez et al., 1994), and regulate blood pressure and cardiac performance *via* activation of the sympathetic nervous system (Marshall, 1994). The CBs apart from participating in cardiorespiratory control are also involved in the homeostatic regulation of carbohydrates, lipid metabolism, and inflammation (Ribeiro et al., 2013; Conde et al., 2017; Conde et al., 2020; Sacramento et al., 2020; Iturriaga et al., 2021). In fact, in the last decade it has become consensual that the CBs are involved in the genesis of metabolic diseases since 1) CB activity is increased in prediabetes and T2D animal models (Ribeiro et al., 2013; Ribeiro et al., 2018) and prediabetic patients (Cunha-Guimaraes et al., 2020); 2) insulin, leptin, and glucagon-like peptide-1 (GLP-1) mediators that contribute to metabolic homeostasis are known to act on the CBs (Ribeiro et al., 2013; Ribeiro et al., 2018; Caballero-Eraso et al., 2019; Cracchiolo et al., 2019; Pauza et al., 2022); and 3) the abolishment of the CB activity, *via* CSN resection or its neuromodulation, prevents and reverses pathological features of dysmetabolism in animal models of dysmetabolism (Ribeiro et al., 2013; Sacramento et al., 2017; Sacramento et al., 2018).

Ageing is known to have led to a marked decrease in CB function, demonstrated by profound changes in CB morphology (Hurst et al., 1985; Di Giulio et al., 2003; Conde et al., 2006; Sacramento et al., 2019), namely, with hyperplasia, decreased number of type I cells (Conde et al., 2006; Sacramento et al., 2019) and progressive proliferation of type II cells (Di Giulio et al., 2003), reduced release of neurotransmitters as catecholamines and adenosine and ATP (Conde et al., 2006; Sacramento et al., 2019), and decreased CSN responses to hypoxia (Conde et al., 2006; Sacramento et al., 2019). Considering the tight link between ageing and metabolic deregulation, the role of CBs in the development of metabolic diseases and the ageing-associated alterations within this organ, as well as the beneficial effects of CSN resection on metabolic dysfunction, herein, we evaluated the effect of long-term hypercaloric diet intake and CSN denervation on age-induced metabolic dysfunction and ventilation. Moreover, we investigated the effect of hypercaloric diet intake, ageing, and CSN resection on the morphological characteristics of the CBs, as well as on hypoxia-inducible factor 1-alpha (HIF-1α) and on insulin receptor and GLP-1 receptor, markers for stimuli that act on the CBs.

## Materials and Methods

### Ethics

All animal experimental and care procedures were approved by the NOVA Medical School|Faculdade de Ciências Médicas (NMS|FCM) Ethics Committee, by the NMS|FCM Animal Welfare Office (ORBEA), and by Direcção-Geral de Veterinária (DGAV), Portugal. The principles of laboratory care were followed in accordance with the European Union Directive for Protection of Vertebrates Used for Experimental and Other Scientific Ends (2010/63/EU). Sample calculation was performed at https://www.stat.ubc.ca/and was based on previous experiments where the effect of carotid sinus nerve resection was tested on metabolic variables assessed *in vivo* in rodents (Ribeiro et al., 2013; Sacramento et al., 2017; Sacramento et al., 2018). A sample size of 6 was defined for both young and adult animals.

### Animals and Surgical Procedures

Experiments were performed on 8-week-old male Wistar rats (200–300 g) obtained from the vivarium of the NOVA Medical School|Faculdade de Ciências Médicas, Universidade Nova de Lisboa, Lisboa, Portugal. The animals were kept under controlled temperature and humidity (21 ± 1°C; 55 ± 10% humidity) with a 12 h light/dark cycle and ad libitum access to food and water. The animals were subjected to either a standard diet (14.53% protein, 10% fat, and 55.06% carbohydrates; RM3, SDS - Special Diet Services, United Kingdom) or to a high-fat high-sucrose diet (HFHSu, 60% lipid-rich diet (23.1% protein, 34.9% fat, and 25.9% carbohydrates; 58Y1, TestDiet, Missouri; United States) with 35% sucrose in drinking water) during 14 or 44 weeks of diet. At 14 or 44 weeks of diet, the animals were randomly divided, and half of the group was subjected to either CSN denervation or to a sham procedure. The rats were anesthetized with ketamine (30 mg/kg, Nimatek, Dechra, Netherlands) and medetomidin (4 mg/kg, Sedator, Dechra, Netherlands), and under aseptic conditions, the carotid artery bifurcations were located bilaterally, and the CSNs were identified and resected bilaterally or left intact (sham) (Ribeiro et al., 2013; Sacramento et al., 2017; Sacramento et al., 2018). Anesthesia was reversed with atipamezole (0.25 mg/kg in 2 ml, i.p., Antisedan, Esteve, Finland), and after the surgery, the animals were treated with analgesic buprenorphine (10 μg/kg, s.c.) and for 2–3 days, with anti-inflammatory carprofen (5 mg/kg, s.c, Rimadyl, Pfizer, Zaventem, Belgium).

Body weight and animal behavioral changes were assessed twice per week. Diet and water consumption were also monitored twice per week. At the end of experiments, the rats were euthanized by an intracardiac overdose of sodium pentobarbital (60 mg/kg i.p.), except when a heart puncture was performed to collect blood.  

### Insulin Tolerance Test

Insulin sensitivity was evaluated *in vivo* by an insulin tolerance test (ITT) (Monzillo and Hamdy, 2003) in conscious animals before the beginning of the diet protocol, after 14 and 44 weeks of diet (before surgery) and 2 and 9 weeks after surgery. After overnight fasting (approx. 16 h), a bolus of insulin (Humulin regular 100 Ul/ml, Lilly) was administered in the tail vein (0.1 U (4.5 g)/kg), and the decline in glycemia was monitored for 15 min with a 1-min interval. Blood samples were collected by the tail tipping method and measured with a glucometer FreeStyle Precision (Abbott Diabetes Care, Portugal) and test strips (Abbott Diabetes Care, Portugal). With the collected data, the constant of the glucose decline rate (K_ITT_), which correlates directly with insulin sensitivity, was calculated using the formula 0,693/(t_1/2_), where glucose half-time (t_1/2_) was calculated from the slope of the curve by the least-square analysis of plasma glucose concentrations during the linear phase decay (Sacramento et al., 2017; Melo et al., 2019).

### Oral Glucose Tolerance Test

Glucose tolerance was evaluated by an oral glucose tolerance test (OGTT) in conscious animals before the beginning of the diet protocol, after 14 and 44 weeks of diet (before surgery) and 9 weeks after surgery, following overnight fasting. For the OGTT, glucose salt solution (2 g/kg) (VWR Chemicals, Leuven, Belgic) was administered by gavage after the measurement of basal glycemia by the tail tipping method. After glucose administration, plasma glucose levels were monitored at 15, 30, 60, 120, and 180 min, as previously described (Sacramento et al., 2017; Melo et al., 2019).

### Plethysmography

Ventilation was measured in conscious freely moving rats by whole-body plethysmography after 14 and 44 weeks of diet (before surgery) and 9 weeks after surgery, as previously described by Sacramento et al. (2018). The system (emka Technologies, Paris, France) consisted of 5-L methacrylate chambers continuously fluxed (2 L/min) with gases. Tidal volume (VT; ml), respiratory frequency (f; breaths/min; bpm), the product of these two variables, and minute ventilation (VE; ml/min/Kg) were monitored. Each rat was placed in the plethysmography chamber and allowed to breathe room air for 20 min until they adapted to the chamber environment and acquired a standard resting behavior. The protocol consisted of subjecting the animals to 30 min acclimatization followed by 10 min of normoxia (20% O_2_ balanced N_2_), 10 min of hypoxia (10% O_2_ balanced N_2_), 10 min of normoxia, 10 min of hypercapnia (20% O_2_ + 5% CO_2_ balanced N_2_), and finally to 10 min of normoxia. A high-gain differential pressure transducer allows measuring the pressure change within the chamber reflecting tidal volume (VT). False pressure fluctuations due to rat movements were rejected. Pressure signals were fed to a computer for visualization and storage for later analysis with EMKA software (emka Technologies, Paris, France).

### Quantification of Plasma Insulin and C-Peptide

Insulin and C-peptide concentrations were determined with an enzyme-linked immunosorbent assay (ELISA) kit (Mercodia Ultrasensitive Rat Insulin ELISA Kit and Mercodia Rat C-peptide ELISA Kit, respectively, Mercodia AB, Uppsala, Sweden).

### Immunohistochemistry Studies

The carotid bifurcation was collected from the animal, and the CBs with a small piece of the carotid artery were bilaterally dissected and immersion-fixed in 4% paraformaldehyde (PFA). The samples were embedded into OCT (Sakura Finetek Europe B.V., Zoeterwoude, Netherlands) and frozen. Serial sections of 8 μm thickness were obtained with a Leica CM3050 S cryostat (Leica Biosystems, Nussloch, Germany). The CB sections were washed with PBS for 5 min and incubated in permeabilizing-blocking solution (PBS containing 0.3% Triton X-100 and 2% non-immunized goat serum) for 20 min at room temperature, as previously described by Sacramento et al. (2019). After blocking, the CB sections were incubated with the primary antibodies rabbit anti-tyrosine hydroxylase (TH) (1:1000, Abcam, Cambridge, United Kingdom) or mouse anti-TH (1:1000, Sigma, Madrid, Spain), glial fibrillary acidic protein (GFAP) (1:250; Dako, Glostrup, Denmark), HIF-1α (1:500, Sicgen, Cantanhede, Portugal), insulin receptor (1:200, Cell Signaling, Madrid, Spain), and GLP-1R (1:500, Abcam, Cambridge, United Kingdom) overnight at 4°C. After washing with PBS (4 × 5 min), the sections were incubated with the secondary antibody goat anti-rabbit Alexa Fluor 594 (1:2000, Abcam, Cambridge, United Kingdom) or chicken anti-goat DyLight 550 (1:1000, Sicgen, Cantanhede, Portugal) or goat anti-mouse Alexa Fluor 488 (1:1000, Invitrigen, Madrid, Spain) or donkey anti-rabbit Alexa Fluor 488 (1:1000, Invitrigen, Madrid, Spain) in a permeabilizing-blocking solution for 1 h at room temperature. The sections were washed with PBS (4 × 5 min) and were incubated with 4,6-diamidino-2-phenylindole (DAPI) (1 μg/ml; Santa Cruz Biotechnology) for 5 min at room temperature. Finally, the CB sections were washed with PBS (2 × 5 min) and mounted with VECTASHIELD (Vector Laboratories, Burlingame, CA, United States). Negative controls were, however, similarly incubated in the absence of a primary antibody. The sections were examined using a fluorescence microscope (ZEISS Axio Imager 2; ZEISS, Oberkochen, Germany) with the excitation and emission filters for Alexa Fluor 594, 488, and 550. Images were captured using Axiocam 105 color (ZEISS) and stored in a computer.

### Data and Statistical Analysis

Hypoxic and hypercapnic ventilatory responses were assessed by quantifying ventilatory parameters, respiratory frequency, tidal volume, and its product minute ventilation during the first 3 min after chamber gas replacement (that correspond approximately to the first 2 min of hypoxic/hypercapnic challenge), thus corresponding to the augmenting phase of the ventilatory response. Analysis of the intensity of fluorescence in immunohistochemistry studies was performed by the piece of software, Fiji app for ImageJ (https://imagej.nih.gov/ij/). TH, GFAP, HIF-1α, insulin receptor, and GLP-1R fluorescence intensity and the entire area of the CB tissue in each section were measured to calculate the percentage of fluorescence intensity per CB area. Each data point in immunofluorescence graphs corresponds to the mean values of serial sections of one CB.

For statistical analyses, data were evaluated using GraphPad Prism software, version 8 (GraphPad Software Inc., United States), and results were expressed as mean ± SEM. The significance of the differences between the mean values was calculated by one- and two-way ANOVA with the Bonferroni multiple comparison test. Differences were considered significant at *p* < 0.05.

## Results

### Effect of Ageing and Long-Term Intake of Hypercaloric Diet on Insulin Sensitivity and Glucose Tolerance

**FIGURE 1:**
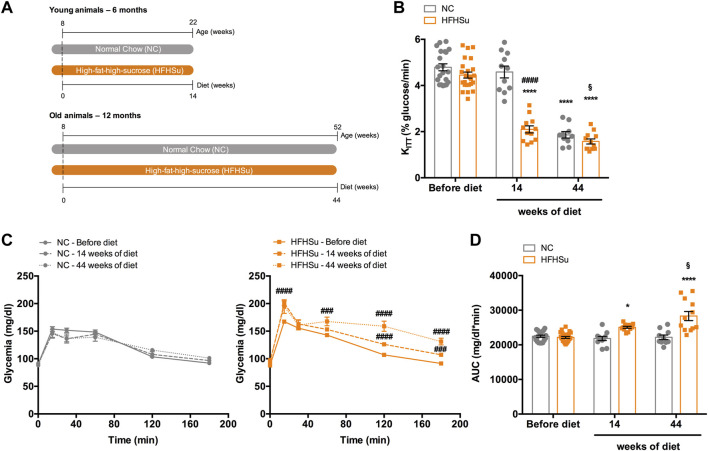
Effect of ageing and long-term intake of hypercaloric diet on insulin sensitivity and glucose tolerance. **(A)** Schematic illustration of the protocol of the study. **(B)** Effect of high-fat high-sucrose (HFHSu) diet on insulin sensitivity assessed by the insulin tolerance test and expressed as the constant of the insulin tolerance test (K_ITT_). **(C)** Effect of long-term intake of high-fat high-sucrose (HFHSu) diet on glucose tolerance depicted as glucose excursion curves in normal chow (NC) and HFHSu animals before diet and 14 and 44 weeks of diet. **(D)** Area under the curve (AUC) obtained through the analysis of the glucose excursion curves. Bars represent mean ± SEM of 10 – 24 animals. One- and two-way ANOVA with Bonferroni multicomparison tests; *p < 0.05 and ****p < 0.0001 *vs*. before diet; ^####^ *p* < 0.0001 compared with normal chow within the same group; ^§^ *p* < 0.05 comparing young *vs*. adult with the same diet treatment.

Figure 1 shows the effect of ageing and hypercaloric diet consumption during 14 and 44 weeks on insulin sensitivity and glucose tolerance, which correspond to animals of 6 and 12 months of age, respectively. As previously described by Guarino et al. (2013), ageing caused a decrease of 61% in insulin sensitivity in normal chow animals (Figure 1B). In animals subjected to 14 and 44 weeks of HFHSu diet, insulin sensitivity also decreased by 53 and 64%, respectively (Figure 1B; K_ITT_ 14 weeks HFHSu = 2.10 ± 0.15 %glucose/min; K_ITT_ 44 weeks HFHSu = 1.57 ± 0.11 %glucose/min), these values being statistically different. Additionally, long-term hypercaloric intake, but not ageing, affects glucose tolerance, as shown in glucose excursion curves (Figure 1C) and in the area under the curve (AUC) obtained from the glucose excursion curves (Figure 1D). It should be noted that hypercaloric diet intake during 14 and 44 weeks increased by 13 and 28% the AUC of the glucose excursion curves (AUC 14 weeks HFHSu = 25052 ± 286 mg/dl*min; AUC 44 weeks HFHSu = 28305 ± 1320 mg/dl*min, *p* < 0.05), respectively (Figure 1D), showing that the deleterious effect on glucose tolerance is as high as the time under hypercaloric diet intake.

### Effect of CSN Resection on Insulin Sensitivity and Glucose Tolerance

**FIGURE 2:**
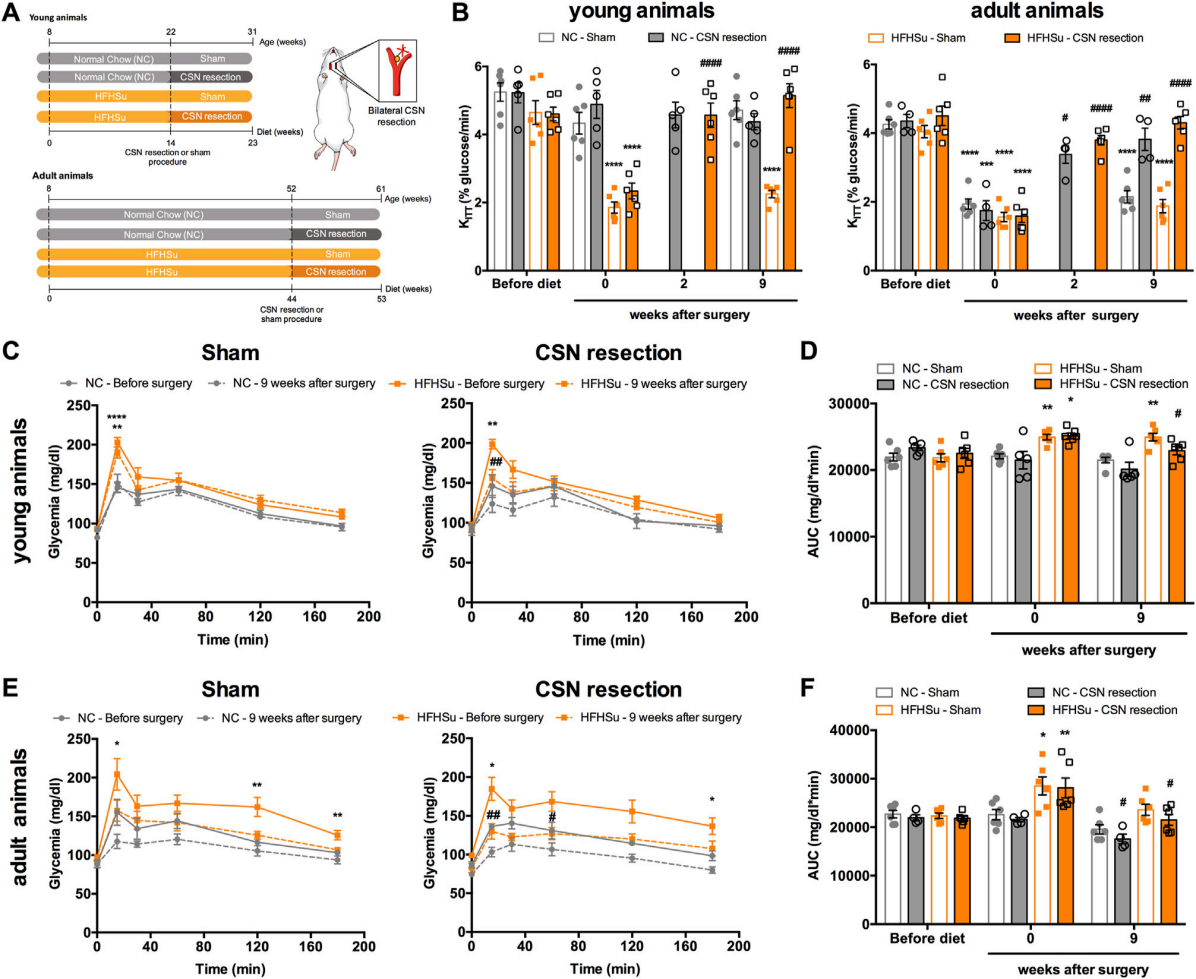
Effect of carotid sinus nerve (CSN) denervation on insulin sensitivity and glucose tolerance in normal chow (NC) and high-fat high-sucrose (HFHSu) animals. **(A)** Schematic illustration of the protocol of the study. **(B)** Effect of CSN resection on insulin sensitivity assessed by the insulin tolerance test and expressed as the constant of the insulin tolerance test (K_ITT_) in normal chow (NC) and HFHSu animals in young and adult animals. **(C)** Effect of sham surgery (left panel) and CSN resection (right panel) on glucose tolerance depicted as glucose excursion curves in NC and HFHSu young animals. **(D)** Area under the curve (AUC) obtained through the analysis of the glucose excursion curves in NC and HFHSu young animals. **(E)** Effect of sham surgery (left panel) and CSN resection (right panel) on glucose tolerance depicted as glucose excursion curves in NC and HFHSu adult animals. **(F)** Area under the curve (AUC) obtained through the analysis of the glucose excursion curves in NC and HFHSu adult animals. Bars represent mean ± SEM of 5–6 animals. One- and two-way ANOVA with Bonferroni multicomparison tests; *p < 0.05, **p < 0.01, and ****p < 0.0001 *vs*. before diet; ^#^ *p* < 0.05, ^##^ *p* < 0.01, and ^####^ *p* < 0.0001 comparing sham *vs*. CSN resection.

Figure 2 shows the effect of chronic bilateral CSN resection on insulin sensitivity and glucose tolerance. As expected and previously described by our group (Sacramento et al., 2018), the CSN resection completely restored insulin sensitivity in young animals subjected to the HFHSu diet, an effect that was observed 2 weeks after the surgery (HFHSu young CSN resection 2 weekspost-CSN resection = 4.57±0.36 %glucose/min) and maintained in the ensuing 9 weeks (HFHSu young CSN resection 9 weeks post-CSN resection = 5.15±0.35 %glucose/min) (Figure 2B) (CTL young 9 weeks post-surgery sham = 4.72±0.24%glucose/min). In HFHSu adult animals submitted to CSN resection, insulin sensitivity was partially restored 2 weeks after the surgery (HFHSu adult CSN resection 2 weeks post-CSN resection = 3.80±0.14 %glucose/min), being completely restored 9 weeks after the surgery (HFHSu adult 9 weeks post-CSN resection = 4.30±0.18 %glucose/min) (Figure 2B). CSN resection also restored almost completely, insulin sensitivity in age-induced insulin-resistant normal chow animals (K_ITT_ NC adult sham 9 weeks post-surgery = 2.14±0.18 %glucose/min; NC adult CSN resection 9 weeks post-surgery = 3.82±0.33 %glucose/min) (Figure 2B). Figures 2C and E show glucose excursion curves of the OGTT in young and adult animals, respectively. Analysis of the AUC of these glucose excursion curves showed that 9 weeks post-CSN resection, glucose intolerance is decreased by 9 and 24% in HFHSu young and adult animals, respectively (Figures 2D, F) (HFHSu young 9 weeks post-CSN resection = 22915 ± 625 mg/dl*min; HFHSu adult 9 weeks post-CSN resection = 21488 ± 1114 mg/dl*min). In normal chow adult animals, CSN resection also decrease the AUC by 19% (Figure 2F) (CTL adult 9 weeks post-CSN resection = 17575 ± 1003 mg/dl*min).

### Effect of Long-Term Hypercaloric Intake, Ageing, and CSN Resection on Fasting Glycemia, Insulinemia, and C-Peptide Levels

Table 1 displays the effect of long-term HFHSu intake, ageing, and CSN resection on fasting glycemia, insulinemia, and C-peptide concentrations in normal chow and HFHSu young and adult animals. Fasting glycemia increased 11% in young HFHSu animals, an effect restored by the CSN resection. In adult animals, long-term HFHSu diet intake, ageing, or CSN resection did not alter fasting glycemia. In contrast and as previously described by Guarino et al. (2013), age increased fasting insulinemia by 439% in normal chow animals. The short- and long-term HFHSu intake also increased plasma insulin levels by 232 and 448%, respectively, when compared with normal chow young animals (Table 1). Nevertheless, the impact of long-term HFHSu consumption and age on insulin levels was similar. Additionally, 9 weeks after the CSN resection, the plasma insulin levels decreased by 45% in HFHSu young animals. However, CSN resection was not able to decrease the hyperinsulinemia induced by age or by the long-term HFHSu consumption (adult animals).

**TABLE 1:** Effect of long-term hypercaloric intake, ageing, and carotid sinus nerve resection on fasting glycemia, insulinemia, and C-peptide levels in normal chow (NC) and high-fat high-sucrose (HFHSu) animals.

	Young				Adult			
	**NC**		**HFHSu**		**NC**		**HFHSu**	
	Sham	CSN Resection	Sham	CSN Resection	Sham	CSN Resection	Sham	CSN Resection
Glycemia (mg/dl)	88.33 ± 2.99	88.20 ± 6.16	98.33 ± 3.47*	87.33 ± 4.08^#^	84.57 ± 6.35	83.75 ± 2.29	87.50 ± 3.96	84.43 ± 4.36
Insulin (pmol/L)	196.67 ± 26.65	167.82 ± 52.59	653.27 ± 131.95***	360.59 ± 32.23^#^	1059.14 ± 14.69****	968.80 ± 102.34	1077.13 ± 21.01****^,§§^	968.30 ± 105.67
C-peptide (pmol/L)	850.19 ± 98.53	749.11 ± 196.23	2025.97 ± 387.16*	1858.58 ± 342.78	2172.15 ± 785.99*	2213.10 ± 131.87	2927.65 ± 421.30***	3824.83 ± 375.81

Data are means ± SEM of 4–6 animals. Young animals were subjected to 23 weeks of HFHSu diet, and adult animals were subjected to 53 weeks of HFHSu diet. One and two-way ANOVA with Bonferroni multicomparison test: *p < 0.05, ***p < 0.001, and ****p < 0.0001 *vs*. normal chow young animals; ^#^ *p* < 0.05, with *vs*. without CSN resection; ^§§^ *p* < 0.01 HFHSu young animals *vs*. HFHSu adult animals

Fasting C-peptide levels also increased by 138 and 244% in young and adult animals subjected to the HFHSu diet, respectively (Table 1), comparing with normal chow young animals, the effects that were not modified by the CSN resection. The increase in plasma C-peptide and insulin levels was as higher as the duration of the HFHSu intake. Also, age increased the C-peptide concentration by 155%, an effect that was not altered by the CSN resection.

### Effect of Long-Term Hypercaloric Intake, Ageing, and CSN Resection on Basal Ventilation and on Ventilatory Responses to Hypoxia and Hypercapnia

Table 2 displays the effect of long-term HFHSu intake, ageing, and CSN resection on basal respiratory frequency, tidal volume, and minute volume. The HFHSu diet increased nonsignificantly respiratory frequency without altering tidal or minute volume, when compared with NC animals. As previously described (Quintero et al., 2016; Sacramento et al., 2019), in general, basal ventilatory parameters were lower in the adult animals when compared to young animals both under NC and HFHSu diets. It should be noted that tidal volume and minute volume were significantly decreased by 32 and by 40% in 14-month-old rats subjected to the NC diet and that the basal respiratory frequency was 26% higher in the young HFHSu animals, compared with 14-month-old HFHSu animals. CSN resection did not modify basal ventilatory parameters significantly (Table 2).

**TABLE 2:** Effect of long-term hypercaloric diet intake and carotid sinus nerve (CSN) resection on basal ventilation parameters: respiratory frequency, tidal volume, and minute volume in normal chow (NC) and high-fat high-sucrose (HFHSu) animals.

		Young					Adult			
		NC		HFHSu		NC			HFHSu	
		Sham	CSN resection	Sham	CSN resection	Sham		CSN resection	Sham	CSN resection
Respiratory frequency (bpm)	Before surgery	97.93 ± 8.99	95.50 ± 15.90	115.72 ± 8.70	115.72 ± 8.70	84.11 ± 4.76		74.61 ± 2.09	85.15 ± 6.93^§^	86.99 ± 8.33^§^
	9 weeks after surgery	89.49 ± 7.37	80.18 ± 6.49	109.64 ± 6.17	105.89 ± 7.00	86.41 ± 1.61		75.01 ± 0.34	97.12 ± 9.85	69.73 ± 3.84^θθ^
Tidal volume (ml/kg)	Before surgery	4.76 ± 0.38	4.68 ± 0.61	4.50 ± 0.38	3.88 ± 0.38	3.25 ± 0.45^§^		3.84 ± 0.37	3.92 ± 0.29	3.69 ± 0.16
	9 weeks after surgery	4.75 ± 0.16	4.04 ± 0.31	4.21 ± 0.34	3.21 ± 0.36	3.40 ± 0.13^θθθ^		3.69 ± 0.12	3.72 ± 0.28	3.51 ± 0.24
Minute volume (ml/minute*kg)	Before surgery	459.66 ± 39.31	447.76 ± 79.80	444.69 ± 34.70	447.65 ± 47.18	274.15 ± 49.66^§^		289.42 ± 20.03	357.17 ± 42.78	342.91 ± 59.87
	9 weeks after surgery	427.66 ± 43.06	323.99 ± 40.69	467.80 ± 59.01	340.46 ± 48.03	294.90 ± 16.44^θ^		284.68 ± 18.25	360.94 ± 49.97	270.49 ± 25.60

Data are means ± SEM of 4–6 animals. Young animals were subjected to 23 weeks (short-term) of HFHSu diet, and adult animals were subjected to 53 weeks (long-term) of HFHSu diet. One and two-way ANOVA with Bonferroni multicomparison test: §*p* < 0.05 young animals *vs*. adult animals before surgery; θp<0.05, θθp<0.01, and θθθp<0.001 young animals *vs*. adult animals 9 weeks after surgery

**FIGURE 3:**
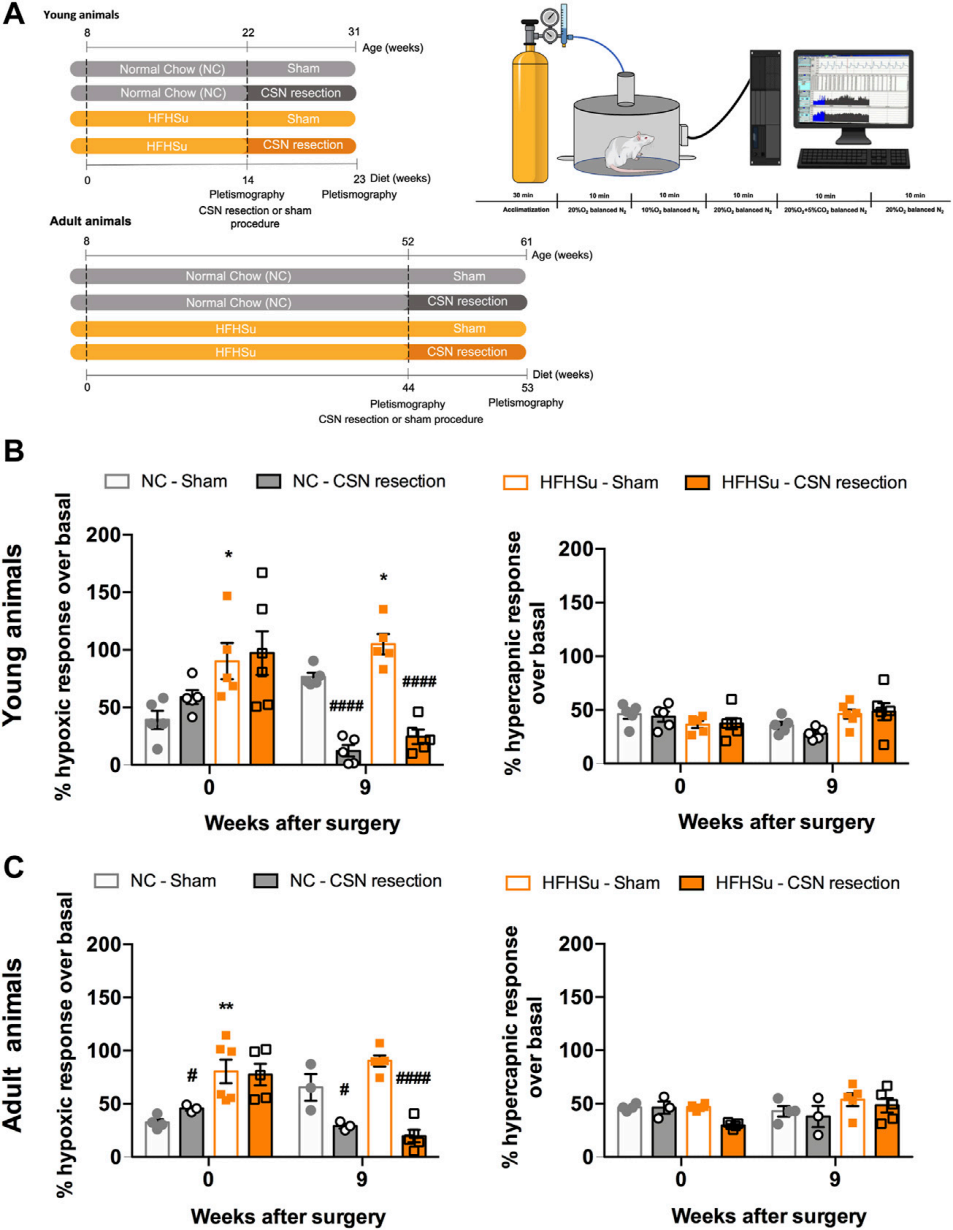
Effect of long-term hypercaloric diet consumption and of carotid sinus nerve denervation on spontaneous ventilation and on the hypoxic and hypercapnic ventilatory responses. **(A)** Left figure shows diet protocols and time points of plethysmographic evaluation. Right figure shows a schematic representation of the protocol used to assess basal ventilation and the ventilatory responses to hypoxia (10% O_2_ balanced N_2_) and hypercapnia (20% O_2_ + 5% CO_2_ balanced N_2_). Acute hypoxia and hypercapnia were applied during 10 min and separated by periods of 10 min in normoxia (20% O_2_). **(B)** Left and right panels show, respectively, the effect of short-term (23 weeks) hypercaloric diet consumption on hypoxic ventilatory and hypercapnic ventilatory responses in young animals. **(C)** Left and right panels show, respectively, the effect of long-term (53 weeks) hypercaloric diet consumption on hypoxic ventilatory and hypercapnic ventilatory responses in young animals, aged rats. Ventilatory responses to acute hypoxia and hypercapnia were represented as minute volume (VE). For each animal and for the entire population of animals, minute ventilation data were normalized to unit body weight. Data from 5 to 6 young animals and from 3–6 for adult rats. Values are mean ± SEM. One- and two-way ANOVA with Bonferroni multicomparison tests; *p < 0.05 and **p < 0.01 *vs*. NC young animals; ^#^ *p* < 0.05 and ^####^ *p* < 0.0001 compared with NC within the same group.

Respiratory responses to hypoxia and hypercapnia are shown in Figure 3. As expected, ageing did not change the ventilatory responses to moderate hypoxia and hypercapnia (Figures 3B, C). In young animals, short-term HFHSu intake increased ventilatory responses to hypoxia by 130% (Figure 3B, left panel) whereas in the adult animals, long-term HFHSu intake increased hypoxia responses by 148% (Figure 3C, left panel). On the other side, CSN resection decreased the ventilatory responses to hypoxia by 79 and 75% in the young NC and HFHSu animals, respectively (Figure 3B, left panel), while in the adult animals, CSN resection decreased the ventilatory responses to hypoxia by 36 and 75% on the NC and HFHSu animals, respectively (Figure 3C, left panel). As expected, no alterations were observed in the ventilatory responses to hypercapnia with HFHSu diet, ageing, or with CSN resection (Figures 3B, C, right panel).

### Effect of Long-Term Hypercaloric Diet Intake and CSN Resection on Type I and Type II Cell Number in the Rat Carotid Body

**FIGURE 4:**
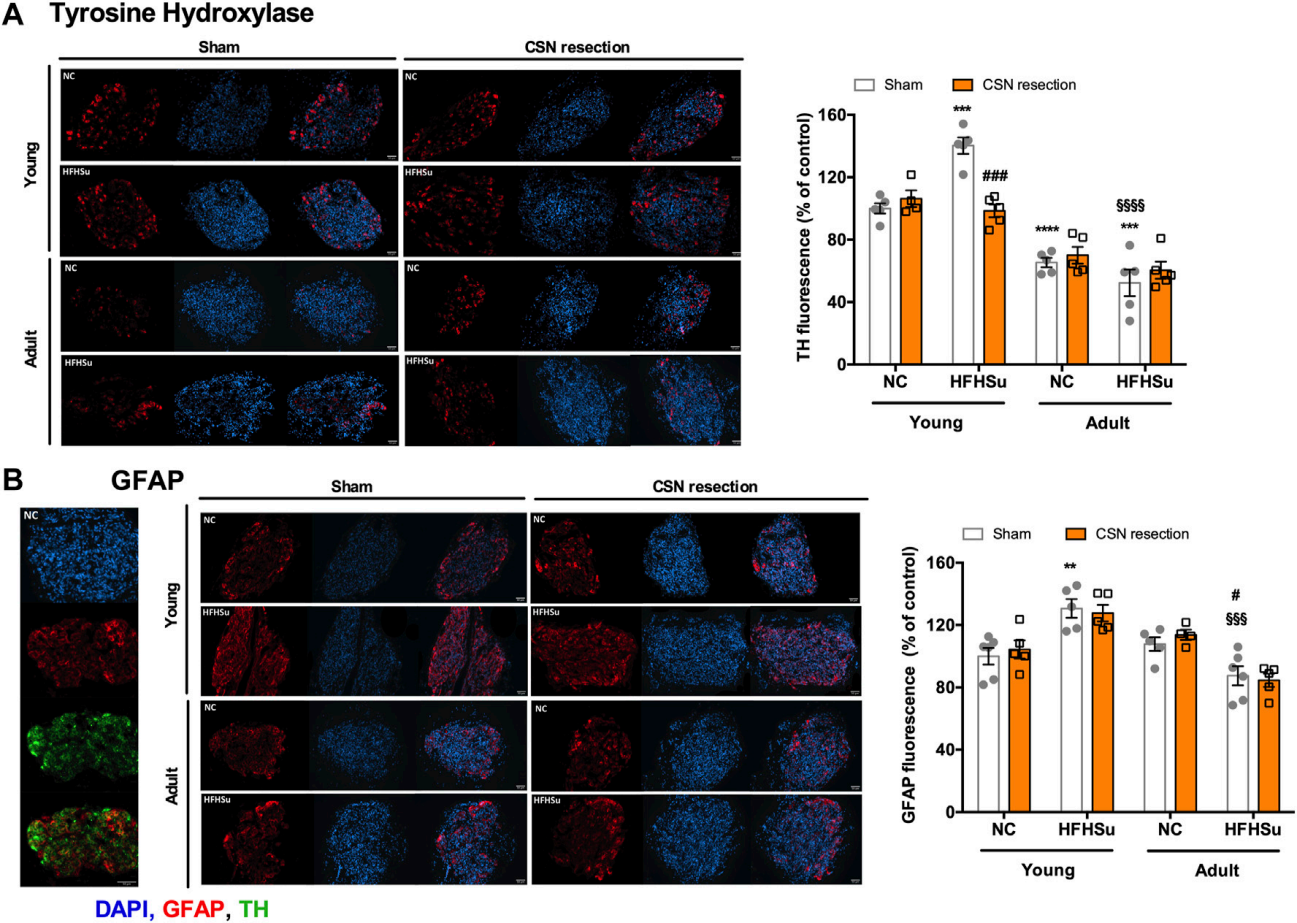
Effect of long-term intake of hypercaloric diet, ageing, and of carotid sinus nerve (CSN) denervation on type I and type II cells in the carotid body (CB). **(A)** Left panel shows immunofluorescence images for tyrosine hydroxylase, a marker for CB type I cells (red fluorescence, left panels) and DAPI (blue fluorescence, middle panels) and its merge (panels on the right) in slices of 7-month-old (young) and 14-month-old (adult) CBs subjected to either a standard or a HFHSu diet with or without CSN resection. The right graph represents mean fluorescence of tyrosine hydroxylase (TH)-positive areas present in serially sectioned CBs. **(B)** Left panel shows typical immunofluorescence images of CB slices from an NC animal with staining for DAPI, a marker of cellular nuclei (blue fluorescence), glial fibrillary acidic protein (GFAP), a marker for CB type II cells (red fluorescence), tyrosine hydroxylase (TH), a marker for type I cells (green fluorescence), and the merge between GFAP and TH. The middle panel shows immunofluorescence images of GFAP (Red fluorescence)- and DAPI (blue fluorescence)-positive staining and its merge in slices of 7-month-old (young) and 14-month-old (adult) CBs subjected to either a standard or a HFHSu diet with or without CSN resection. The right graph shows mean fluorescence of GFAP-positive areas present in serially sectioned CBs. Data from 5 to 6 CBs for young animals and from 4 to 5 CBs for adult rats. Data represent means ± SEM. One- and two-way ANOVA with Bonferroni multicomparison tests; **p < 0.01, ***p < 0.001, and ****p < 0.0001 *vs*. NC young animals; ^#^ *p* < 0.05 and ^###^ *p* < 0.001 comparing sham *vs*. CSN resection within the same group; ^§§§^ *p* < 0.001 and ^§§§§^ *p* < 0.0001 comparing young animals *vs*. adult animals with the same diet treatment.

Figure 4 shows the effects of long-term HFHSu consumption and CSN resection in CB morphology. In accordance with Dos Santos et al. (2018), short-term HFHSu intake increased by 40% of the TH immunoreactivity, a marker for type I cells, which is concomitant with the increase in the CB size observed in prediabetes animal models (Ribeiro et al., 2013) and type 2 diabetes patients (Cramer et al., 2014), an effect that was restored by the CSN resection (Figure 4A). In adult animals, a decrease of 35% was observed in the TH immunoreactivity, representing a decrease in CB type I cells induced by ageing (Figure 4A), as previously described in other studies (Di Giulio et al., 2003; Conde et al., 2006; Sacramento et al., 2019). Long-term HFHSu intake did not modify the TH-positive immunostaining which means that in adult animals the number of type I cells is not modified by the hypercaloric diet intake. No alterations were observed with the CSN resection in adult NC and HFHSu animals (Figure 4A).

In young animals, the HFHSu diet also increased by 31% the percentage of cells immunoreactive to GFAP, a marker for type II cells (Figure 4B), an effect that was not altered by the CSN resection. In adult animals, ageing did not change the immunoreactivity for GFAP, as in accordance with Sacramento et al. (2019). However, long-term HFHSu intake decreased by 19% type II cells, when compared with age-matched NC animals. The CSN resection did not change the percentage of cells immunoreactive to GFAP in both NC and HFHSu adult animals (Figure 4B).

### Long-Term Hypercaloric Diet, Ageing, and CSN Resection Do Not Change HIF-1alpha Levels in the Carotid Body

**FIGURE 5:**
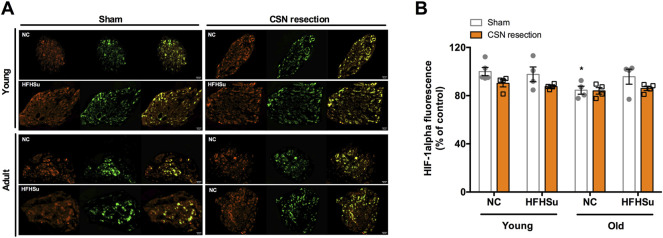
Effect of long-term hypercaloric diet intake, ageing, and of carotid sinus nerve (CSN) resection on HIF-1α staining in the carotid body (CB). **(A)** Panel shows immunofluorescence images for positive staining for HIF-1α (orange fluorescence, left panels) and tyrosine hydroxylase (TH), a marker of CB type I cells, (green fluorescence, middle panels) and its merge (panels on the right) in slices of 7-month-old (young) and 14-month-old (adult) CBs subjected to either a standard or a HFHSu diet with or without CSN resection. (B) Bar graph represents mean fluorescence of HIF-1α-positive areas present in serially sectioned CBs. Data from 4 to 5 CBs for young animals and from 4 CBs for adult rats. Data represent means ± SEM. One- and two-way ANOVA with Bonferroni multicomparison tests; *p < 0.05 *vs*. NC young animals.

Figure 5 shows the effect of HFHSu diet, ageing, and CSN denervation in the hypoxia marker HIF-1α in the CBs. The HIF-1α immunoreactivity (orange fluorescence) is observed in type I cells, as indicated by the colocalization with TH, but also in other cells in the CBs. Ageing decreased by 16% HIF-1α immunoreactivity in NC animals. Additionally, neither HFHSu consumption nor CSN resection changes the percentage of HIF-1α-positive cells in the CBs.

### Effect of Long-Term Hypercaloric Diet Intake, Ageing, and CSN Resection on Receptors of Metabolic Mediators That Drive CB Dysfunction

**FIGURE 6:**
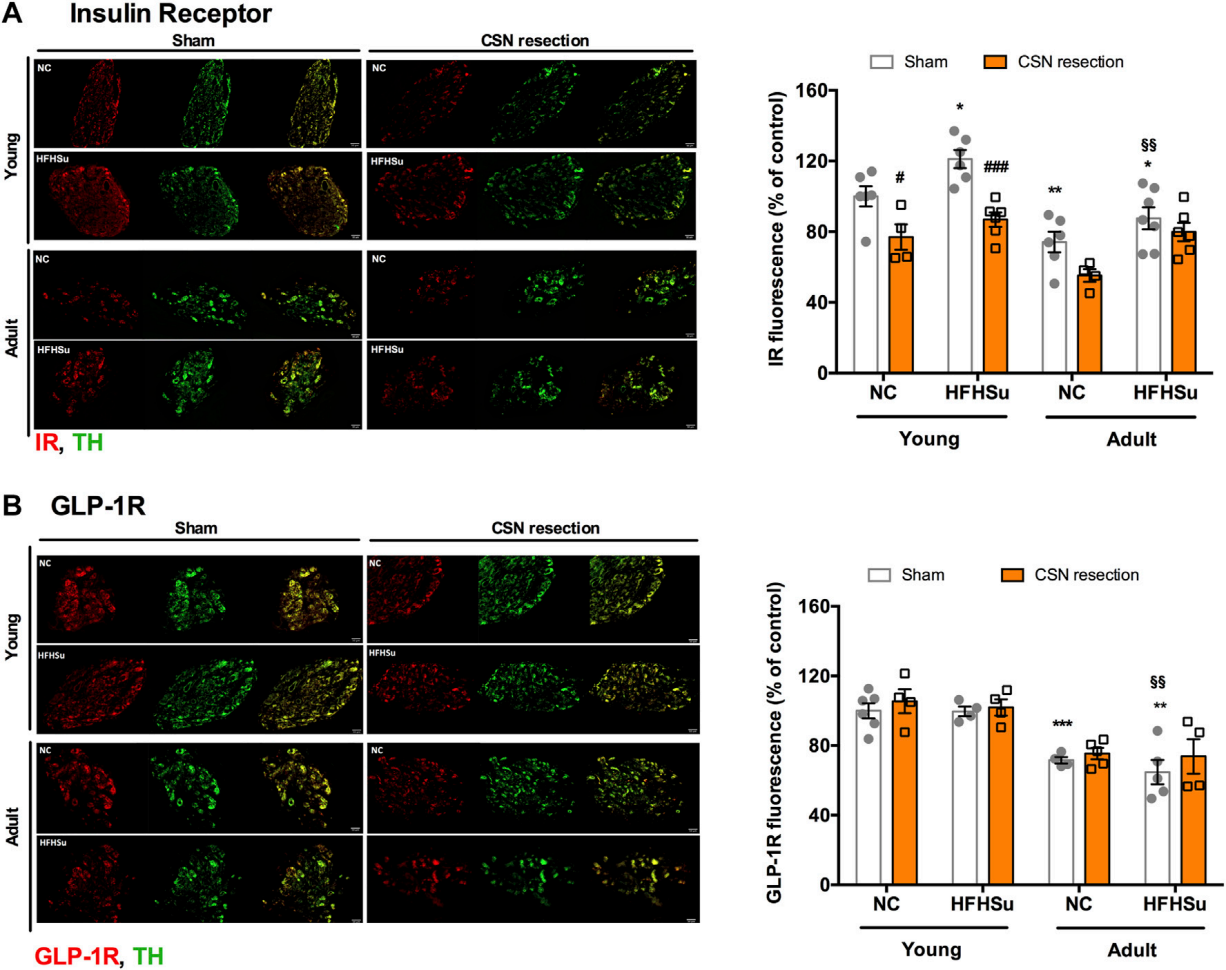
Effect of long-term hypercaloric diet intake, ageing, and carotid sinus nerve (CSN) resection on insulin receptor and glucagon-like peptide-1 receptor (GLP-1R). **(A)** Left panel shows immunofluorescence images for positive staining for the insulin receptor (IR) (red fluorescence, left panels) and tyrosine hydroxylase (TH), a marker of CB type I cells, (green fluorescence, middle panels) and its merge (panels on the right) in slices of 7-month-old (young) and 14-month-old (adult) CBs subjected to either a standard or a HFHSu diet with or without CSN resection. The right graph represents mean fluorescence of IR-positive areas present in serially sectioned CBs. **(B)** Left panel shows immunofluorescence images for the positive staining of GLP-1 receptor (GLP-1R) (red fluorescence, left panels) and TH, a marker of CB type I cells, (green fluorescence, middle panels) and its merge (panels on the right) in slices of 7-month-old (young) and 14-month-old (adult) CBs subjected to either a standard or a HFHSu diet with or without CSN resection. The right graph shows mean fluorescence of GLP1-R-positive areas present in serially sectioned CBs. Data from 4 to 6 CBs for young animals and from 4 to 7 CBs for adult rats. Data represent means ± SEM. One- and two-way ANOVA with Bonferroni multicomparison tests; *p < 0.05, **p < 0.01*,* and ***p < 0.001 *vs*. NC young sham; ^#^ *p* < 0.05 and ^###^ *p* < 0.001 comparing sham *vs*. CSN denervation within the same group; ^§§^ *p* < 0.01 comparing young animals *vs*. adult animals with the same diet treatment.

The CB has been described as a metabolic sensor, and its dysfunction has been associated with metabolic diseases, such as prediabetes and type 2 diabetes (Ribeiro et al., 2013; Conde et al., 2017; Conde et al., 2018; Sacramento et al., 2020). Knowing that insulin (Ribeiro et al., 2013) and GLP-1 (Pauza et al., 2022) are metabolic mediators that activate the CBs, we evaluated the effect of HFHSu consumption, ageing, and CSN resection on the immunoreactivity of insulin receptors and GLP-1R in the CBs (Figure 6). The presence of insulin receptors in the CBs was already described by our group (Ribeiro et al., 2013), but herein, we showed that the insulin receptors are detected in the TH-positive glomus cells (red fluorescence – IR, green fluorescence – TH, Figure 6A). Short-term HFHSu diet intake in young animals increased by 21% the immunoreactivity insulin receptors (Figure 6A)—an effect that was restored by the CSN resection. In young NC animals, the CSN resection also decreased by 23% the percentage of cells immunoreactive to insulin receptors. Moreover, ageing itself decreased the immunoreactivity to insulin receptors by 26%, an effect that decreased non-significantly by 25% in NC adult animals subjected to the CSN resection (Figure 6A). Long-term hypercaloric intake did not modify the effect of ageing on the immunoreactivity of insulin receptors, an effect not altered by the CSN resection (Figure 6A).

Figure 6B presents the immunoreactivity of GLP-1R in young and adult animals and the impact of HFHSu on these levels. It should be noted that GLP-1R (red fluorescence) is detected in type I cells and demonstrated here by the colocalization with TH (green fluorescence), in accordance with Pauza et al. (2022). Short-term HFHSu intake or CSN resection in young animals did not modify the percentage of cells immunoreactive to GLP-1R. In contrast, in NC adult animals, GLP-1R immunoreactivity decreased by 28%. Long-term HFHSu diet consumption did not change the effect of ageing on GLP-1R immunoreactivity. Additionally, no alterations were observed with the CSN resection in adult animals (Figure 6B).

## Discussion

Herein, we found that ageing promoted insulin resistance and increased insulin secretion and that long-term hypercaloric diet intake exacerbates dysmetabolic states, characterized by insulin resistance, glucose intolerance, and increased insulin secretion, effects reversed by CSN denervation. Together, these findings support a potential role for the modulation of CB activity not only for the treatment of early stages of dysmetabolism but also in exacerbated stages of metabolic dysfunction.

We also confirmed that ageing and short-term and long-term hypercaloric diet intake altered basal ventilation and that hypercaloric diet intake exacerbated hypoxic ventilatory responses. We also provide further evidence that dysmetabolic states in young animals are associated with morphological changes in the CBs, characterized by the increased number of type I and II cells, and with increased staining of IR in the CBs, and in old animals, by decreases in IR and GLP-1R staining. Interestingly, CSN resection decreased TH-positive staining in HFHSu animals and IR-positive staining in both NC young and old animals and in HFHSu young animals. Neither hypercaloric diet intake nor CSN resection changed HIF-1α-positive staining in the CBs, suggesting that hypoxia did not play a role in stimulating the CBs in metabolic disease.

### Effect of Long-Term Hypercaloric Diet and CSN Denervation on Whole-Body Insulin Action, Glucose Tolerance, and Insulin Secretion

Herein, we showed that ageing promoted insulin resistance that is accompanied by increased insulin secretion. Additionally, we found that long-term hypercaloric diet intake exacerbated metabolic dysfunction showed by an exacerbated glucose intolerance and insulinemia in comparison with animals subjected to short-term HFHSu diet intake. Our results on the effects of ageing on insulin action and secretion agree with those of our previous work (Guarino et al., 2013) in which we showed that animals aged 12 and 24 months exhibited insulin resistance together with hyperinsulinemia and with several animal (Reaven et al., 1983; Escrivá et al., 2007; Marmentini et al., 2021) and human (Minaker et al., 1982; Fink et al., 1985; Chang and Halter, 2003) studies showing that insulin secretion, insulin clearance, and the interaction between insulin and its target tissues are defective in elderly individuals. Also, we showed that hypercaloric diets promoted a state of metabolic deregulation, characterized by hyperglycemia, increased insulin secretion, insulin resistance, and glucose intolerance (Sacramento et al., 2017; Sacramento et al., 2018; Melo et al., 2019) that is exacerbated by long-term intake. Interestingly, we show here for the first time that CSN resection can restore insulin sensitivity in adult/aged animals without modifying insulin secretion. In agreement with our previous work (Sacramento et al., 2017; Sacramento et al., 2018), CSN resection reversed insulin resistance and attenuated increased insulin secretion and glucose intolerance promoted by short-term hypercaloric diets, but also reversed the exacerbated dysmetabolic states induced by long-term HFHSu intake, without altering insulin secretion. Altogether, these results mean that CSN modulation is capable of ameliorating whole-body metabolism not only in the early stages of metabolic dysfunction but also in states of exacerbated metabolic deregulation, where some of the defective functions are irreversible. Surprisingly, although CSN resection was able to restore insulin action and glucose homeostasis in young and adult/old animals, the fact that this procedure is able to ameliorate insulin secretion in young animals, as previously described (Sacramento et al., 2017), but not in old animals suggest the existence of different mechanisms or different contributions of the same mechanism behind the restoration of metabolism. Knowing that the CB controls peripheral insulin sensitivity (Ribeiro et al., 2013), we can postulate that restoration of insulin action and glucose tolerance in adults subjected to long-term hypercaloric diets might be related to increased insulin action on the target tissues; however, this requires further clarification. Moreover, we also know that ageing and hypercaloric diet consumption are associated with a progressive state of low inflammation (Pawelec et al., 2014) and that the CB is activated by inflammatory cytokines (Fernandez et al., 2014; Conde et al., 2020; Iturriaga et al., 2021), also being involved in the anti-inflammatory reflex (Fernandez et al., 2014; Conde et al., 2020; Iturriaga et al., 2021; Katayama et al., 2022). Therefore, we can postulate that CSN denervation might decrease the inflammatory state and therefore ameliorate metabolism.

### Effect of Ageing and Long-Term Hypercaloric Diet on Spontaneous Ventilation and on Ventilatory Responses to Hypoxia and Hypercapnia

As we previously described (Quintero et al., 2016; Sacramento et al., 2019), herein, we found that 14-month-old rats exhibited decreased tidal volume resulting in lower minute ventilation. However, this decrease in basal ventilation does not mean hypoventilation as it is described that ageing is accompanied by a decrease in whole-body O_2_ consumption (Cerveri et al., 1995; Chan and Welsh, 1998). Moreover, we found that both short-term and long-term hypercaloric diet intakes promoted a tendency to alter ventilatory parameters, with short-term hypercaloric diet increasing the basal ventilatory frequency in young animals and long-term diet inducing increased tidal volume in adult animals. Although we might think that HFHSu animals would have an obesity hypoventilatory syndrome due to obesity (Masa et al., 2019), these animals do not exhibit a weight gain compared with other dysmetabolic/obese animals, for example, animals subjected to a high-fat diet or the zucker fatty diabetic animals (Melo et al., 2019; Baby et al., 2022). Herein, as previously described, we showed that ageing does not change hypoxic ventilatory responses to moderate hypoxia (10% O_2_); however, HFHSu diet potentiated this response in both young and adult animals, suggesting that hypercaloric diets alter CB chemoresponses to hypoxia and probably its integration within the central nervous system. These results are not new as in the past, we showed that the ventilatory responses to the occlusion of the common carotid artery (ischemic hypoxia) were increased in high-fat diet animals (Ribeiro et al., 2013), and we attributed these effects to the effect of insulin on CBs. Knowing that insulin activates the CBs (Ribeiro et al., 2013; Cracchiolo et al., 2019) and increases ventilation, an effect described in animals (Bin-Jaliah et al., 2004; Ribeiro et al., 2013) and in humans (Ward et al., 2007; Barbosa et al., 2018), it is not strange that HFHSu animals that are hyperinsulinemic could exhibit increased hypoxic ventilatory responses. In agreement with the role of CBs in mediating hypoxic responses (Gonzalez et al., 1994) and with the role of insulin in CBs (Conde et al., 2018), CSN resection abolishes the ventilatory responses to hypoxia almost entirely.

### Effect of Long-Term Hypercaloric Diet, Ageing, and CSN Denervation on CB Morphology and on Markers for Key Stimuli That Activate the CB

In an attempt to correlate the effects of short-term and long-term hypercaloric diet intake, ageing, and of CSN resection on whole-body metabolic parameters with CB function, we analyzed, by immunohistochemistry, markers of type I and type II cells of the CBs, the TH, and GFAP, as well as a marker for hypoxia, HIF-1α, and receptors for metabolic mediators that are known to act on the CBs, the insulin receptor (Ribeiro et al., 2013) and GLP-1R (Pauza et al., 2022). As expected, as previously described by our group and others, adult/old CBs exhibited less positive staining for TH, this corresponding to a lower number of type I cells per CB area (Di Giulio et al., 2003; Conde et al., 2006; Sacramento et al., 2019), thus agreeing with the lower baseline ventilatory parameters found in adult animals. Also, in agreement with our studies showing an overactivation of the CBs in metabolic disease states (Ribeiro et al., 2013; Dos Santos et al., 2018; Ribeiro et al., 2018; Cunha-Guimaraes et al., 2020) and with the work of Cramer that found that CBs from type 2 diabetic patients are 25% bigger than those of healthy individuals (Cramer et al., 2014); herein, we observed that short-term HFHSu diet intake significantly increases the number of TH-positive and GFAP-positive staining, suggesting that these increased number of cells might contribute to an increased function of this organ. We can hypothesize that this increase in type I and II CB cells can be due to the action of high insulin circulating levels, present in HFHSu animals, in promoting cell proliferation. This agrees with the effects of insulin in inducing cell proliferation in white adipose tissue (Géloën et al., 1989), in beta-pancreatic cells *in vivo* (Beith et al., 2008), and in several cell lines in culture (Heni et al., 2011). CSN denervation by acting to restore insulin secretion could impact type I and II CB cell numbers. In contrast, long-term hypercaloric diet intake was unable to modify TH-positive staining and decreased even more the GFAP-positive staining in comparison with young and old CBs, suggesting that in ageing and with exposure to long-term HFHSu diet, the number of type I cells does not determine the level of CB dysfunction. However, we can suggest that the decrease in type II cells promoted by a long-term hypercaloric diet might be involved in the dysfunction of the CBs in ageing. It is known that CB type II cells are multipotent stem cells involved in CB neuroplasticity and that can be differentiated in other cell types (Pardal et al., 2007; Sobrino et al., 2020) as mesenchyme-like vascular cells, such as pericytes, smooth muscle, or even endothelial cells, as it has already been described (Navarro-Guerrero et al., 2016; Sobrino et al., 2020), which display no signs of neuronal (TH-positive cell) differentiation. Therefore, we can speculate that the loss of GFAP-positive staining in the CBs of animals submitted to long-term HFHSu intake might be due to the differentiation of type II cells in other cell types than type I cells, which could contribute to metabolic dysfunction, although these contentions require further clarification.

One of the stimuli that activates the CB and that promotes alterations in its chemosensory activity and in cell proliferation is hypoxia (Gonzalez et al., 1994; Sobrino et al., 2020). Also, chronic hypoxia is usually associated with increased weight gain and metabolic dysfunction (Norouzirad et al., 2017), and therefore, we explored if hypoxia within the CBs could be contributing to CB dysfunction and metabolic deregulation induced by hypercaloric diet intake. Herein, we did not find any alterations in HIF-1α immunofluorescence–positive staining within the CB (analyzed per CB area), induced by short- or long-term HFHSu intake, suggesting that hypoxia within the CB is not involved in the mechanisms promoting CB dysfunction and metabolic deregulation in our animals. Interestingly, we also found that ageing itself decreased HIF-1α immunofluorescence staining within the CBs. The absence of hypoxia, herein, observed indirectly by assessing HIF-1α immunofluorescence in the CBs together with the enlargement of CBs promoted age (Conde et al., 2006; Sacramento et al., 2019) and hypercaloric diets (Ribeiro et al., 2013), suggest that in these situations, correct mechanisms of angiogenesis are being put in place to allow the correct delivery of O_2_ to the tissue.

Insulin and GLP-1 are metabolic mediators that are known to act on the CBs and whose activity in this organ has been postulated to be involved in metabolic dysfunction (Ribeiro et al., 2013; Conde et al., 2018; Cracchiolo et al., 2019; Pauza et al., 2022). Herein, we confirmed that IR and GLP-1R, receptors for insulin and GLP-1, colocalize with tyrosine hydroxylase in type I cells of the CBs. As expected, IR-positive staining in the CBs was significantly increased in animals subjected to short-term HFHSu intake and slightly increased in animals subjected to long-term HFHSu diet in comparison with their respective controls, thus agreeing with the overactivation of the CBs found in animal models of dysmetabolism (Ribeiro et al., 2018; Cracchiolo et al., 2019) and prediabetic patients (Cunha-Guimaraes et al., 2020). In fact, CB chemosensitivity in prediabetic patients was shown to be correlated with insulin levels and with insulin resistance (Cunha-Guimaraes et al., 2020). The increase in IR staining in young animals was also in agreement with the enlargement of the CBs in metabolic disease animals (Ribeiro et al., 2013) and patients (Cramer et al., 2014) and with the increase in the number of type I cells in these CBs herein observed and with the increased CSN activity in HFHSu young animals and response to insulin (Cracchiolo et al., 2019), clearly stating that insulin in early stages of metabolic dysfunction contributes to CB dysfunction. CBs from adults/old animals showed lower IR staining per CB area, reflecting the decreased TH-positive staining area. Interestingly, as stated before, long-term HFHSu intake slightly increases IR staining, suggesting that even less TH-positive insulin seems to contribute to CB dysfunction. In agreement with the effect of circulating insulin on the CBs, CSN denervation led to a decrease in IR immunofluorescence staining in the CBs in all groups of animals, except the long-term HFHSu group of animals. This decrease in IR staining in the CBs clearly reflects the improvement in insulin secretion and the amelioration of peripheral metabolism promoted by CSN denervation observed herein and previously described (Sacramento et al., 2017; Sacramento et al., 2018).

Surprisingly, neither short-term or long-term HFHSu diet nor even CSN resection alter GLP1-R staining, suggesting that this mediator is not involved in CBs and metabolic dysfunction promoted by hypercaloric diets. However, if we take into consideration that HFHSu animals subjected to a short-term hypercaloric diet exhibit an increased number of TH cells, this probably means that GLP-1R will be less expressed in type I cells, therefore contributing to CB dysfunction. This agrees with the findings of Pauza et al. (2022), where they showed that CBs from spontaneously hypertensive rats exhibited a decrease in the expression of GLP-1 receptors that is associated with increased chemoreflex-evoked sympathetic drive. However, the absence of effects of long-term hypercaloric diet intake and of CSN resection clearly led to the idea that further research is needed to understand the contribution of GLP-1 and GLP-1R to dysmetabolic states induced by hypercaloric diets, those that better mimic human metabolic diseases, such as obesity and type 2 diabetes.

## Conclusion Remarks

In conclusion, long-term hypercaloric diet consumption exacerbates age-induced dysmetabolism, and both short and long-term hypercaloric diet intake promote significant alterations in CB function, seen by alterations in type I and type II cell numbers, as well as by significant increases in insulin receptor staining. CSN resection ameliorates these effects, suggesting that the modulation of CB activity apart from being beneficial in initial states of dysmetabolism as in prediabetes might also have a significant impact on exacerbated stages of dysmetabolism.

The fact that we had obtained different results on the CBs of young and adult animals clearly highlights the importance of studying animals of different ages and in different stages of disease progression as the mechanisms involved in metabolic deregulation might be different or have different contributions.

## Data Availability

The raw data supporting the conclusions of this article will be made available by the authors, without undue reservation.
